# Participatory survey of Rift Valley fever in nomadic pastoral communities of North-central Nigeria: The associated risk pathways and factors

**DOI:** 10.1371/journal.pntd.0006858

**Published:** 2018-10-30

**Authors:** Nma Bida Alhaji, Olutayo Olajide Babalobi, Yiltawe Wungak, Hussaini Gulak Ularamu

**Affiliations:** 1 Public Health and Epidemiology Department, Niger State Ministry of Livestock and Fisheries, Minna, Nigeria; 2 Department of Veterinary Public Health and Preventive Medicine, University of Ibadan, Ibadan, Nigeria; 3 National Veterinary Research Institute, Vom, Nigeria; CDC, UNITED STATES

## Abstract

**Background:**

Rift Valley fever (RVF) is an emerging neglected mosquito-borne viral zoonotic disease of domestic animals and humans, with potential for global expansion. The objectives of this study were: to assess perceived relative burden and seasonality of RVF in nomadic cattle herds and validate the burden with sero-prevalence impact; and assess perceived risk factors associated with the disease and risk pathways for RVF virus in nomadic pastoral herds of North-central Nigeria using pastoralists’ existing veterinary knowledge.

**Methods:**

Participatory Epidemiology (PE) survey was conducted in Fulani nomadic pastoral communities domiciled in Niger State between January and December 2015. A cross-sectional sero-prevalence investigation was also carried out in nomadic pastoral cattle herds to validate outcomes of PE. A total of nine nomadic pastoral communities were purposively selected for qualitative impact assessment using Participatory Rural Appraisal tools, while 97 cattle randomly sampled from 15 purposively selected nomadic herds and had their sera analyzed using c-ELISA. Kendall’s Coefficient of Concordance W statistics and OpenEpi 2.3.1 were used for statistical analyses.

**Results:**

Mean proportional piles (relative burden) of RVF (*Gabi-gabi*^*F*^) was 8.3%, and nomads agreement on the burden was strong (W = 0.6855) and statistically significant (P<0.001). This was validated by 11.3% (11/97; 95% CI: 6.1–18.9) sero-positivity (quantitative impact). Mean matrix scores of prominent clinical signs associated with RVF were fever (3.1), anorexia (2.1), abortion (4.1), nasal discharge (3.3), neurological disorder (8.4), diarrhoea (3.2), and sudden death (4.4), with strong agreement (W = 0.6687) and statistically significant (p<0.001). Mean proportional piles of pastoralists’ perceived risk factors identified to influenced RVF occurrence were: availability of mosquitoes (18 piles, 17.6%), high cattle density (16 piles, 15.9%) and high rainfall (12 piles, 12.2%). Agreement on the risk factors was strong (W = 0.8372) and statistically significant (p<0.01). Mean matrix scores for the Entry pathway of RVF virus into the nomadic pastoral herds were: presence of RVFV infected mosquitoes (tiny biting flies) (7.9), presence of infected cattle in herds (8.4), and contacts of herd with infected wild animals at grazing (10.1). Mean matrix scores for the Spread pathway of RVF virus in herds were bites of infected mosquitoes (5.1), contacts with infected aborted fetuses/fluids (7.8), and contaminated pasture with aborted fetuses/fluids (9.7). Agreement on risk pathways was strong (W = 0.6922) and statistically significant (p<0.03). Key informants scored RVF to occurred more in *Damina* or late rainy season (5.3), followed by *Kaka* or early dry season (3.3), with strong agreement (W = 0.8719) and statistically significant (P<0.01). This study highlighted the significant existing knowledge level about RVF contained in nomadic pastoralists.

**Conclusions:**

The use of PE approach is needful in active surveillance of livestock diseases in pastoral communities domiciled in highly remote areas. RVF surveillance system, control and prevention programmes that take the identified risk factors and pathways into consideration will be beneficial to the livestock industry in Nigeria, and indeed Africa. An ‘OneHealth’ approach is needed to improve efficiency of RVF research, surveillance, prevention and control systems, so as to assure food security and public health in developing countries.

## Author summary

Rift Valley fever (RVF), is a neglected vector-borne zoonotic disease, commonly occurring in Africa and the Arabian Peninsula. It causes severe socio-economic and public health impacts on livestock, humans and environment, placing further economic demands on pastoralist communities already experiencing high level of poverty. There is urgent need to develop new surveillance and control approaches that will identify and tackle RVF in under-privileged communities. One such approach would be by use of Participatory Epidemiology, which involves local communities in the surveillance of diseases, empowering them as agents of change rather than relegating them to disease information providers. RVF is a disease with global implication, but it starts at local level. Therefore, to control this neglected disease, it is important to understand exiting veterinary knowledge contained in the local communities. Using a bottom-up perspective, we investigated factors that influence RVF occurrence and pathways for its entry, spread and consequences in herds. By devising acceptable and cost-effective methods of investigations, we believe pastoralist communities can be encouraged to be the first line of defense against RVF and other neglected animal diseases. Furthermore, policies aimed at preventing RVF occurrence in animals and humans would be beneficiary if pastoral communities are fully involved.

## Introduction

Rift Valley fever (RVF) is an emerging vector-borne viral zoonotic disease of domestic animals and humans [1], caused by the Rift Valley fever virus (RVFV), a member of the Genus *Phlebovirus*, Order *Bunyavirales*, and Family *Phenuiviridae* [2,3]. RVFV is transmitted among domestic and wild ruminants by bites of several species of mosquitoes belonging to the *Aedes* and *Culex* genera and by direct contact with body fluids of viremic animals [4,5]. Moreover, biological or mechanical transmission of RVFV has been reproduced experimentally with other hematophagous arthropods, such as *Culicoides* (biting midges), *Amblyomma* (bont ticks), *Phlebotomus* (sand flies), *Stomoxys* (stable flies), *Glossina morsitans* (Tsetse flies) and *Simulium* (black flies), but field relevance of these transmission routes are still not clear [6,7]. Humans are mainly infected by close contacts with blood, excreta of infected animals, consumption of raw meat and milk [8,9,10,], and in some rare cases, through mosquito bites [11].

Outbreaks of RVF are closely associated with periods of heavy rains and flooding, which increase habitat suitability for vector populations. These factors can drive vector abundance and population dynamics, thus influencing the risk of disease emergence, transmission and spread [12,13]. Movements of viremic animals along trade and cattle routes have been suspected to be responsible for the RVFV spreading [14].

RVF is listed as an emerging zoonotic category A viral pathogen in the National Institute for Allergy and Infectious Diseases (NIAID) list priority pathogens for bio-defense research [5,15]. The disease has a severe socio-economic impact on food security, household nutrition, and trade to livestock producers in affected countries [16,17]. In Kenya, the RVF epidemic that occurred in 2006/2007 caused estimated losses of more than 32 million USD to the economy and extended to various stakeholders in the marketing chain [16]. It is a threat to human health, animal health and livestock production in Africa, the Near East and potentially, Europe and the rest of the world can be affected [13].

Occurrence of large outbreaks of RVF in parts of Africa and Arabian Peninsula has increased the virological and entomological awareness regarding the disease [11,12,18,19,20]. However, understanding the emergence and subsequent spread of emerging infectious diseases is a critical global challenge, especially for high impact zoonotic and vector-borne diseases like RVF. Effective surveillance system and early warning for timely outbreak response of RVF require livestock farmers to have adequate knowledge to detect the disease in advance [11,21]. Studies conducted elsewhere revealed low knowledge of livestock owners following RVF outbreaks. A study conducted in Sudan revealed that 82% of livestock owners did not know modes of transmission and 70% could not identify the correct vector for RVF [21]. Other studies done among agro-pastoralist communities in Kenya and Tanzania showed limited knowledge about RVF signs and symptoms in both animals and humans [18].

Nomadic pastoral communities in Africa live in some of the most underdeveloped environments in the world [22]. Although these communities are reliant on their livestock as a source of socio-economic well-being, conventional veterinary services are poor and basic information on the epidemiology of important livestock diseases is limited. Epidemiological research and disease surveillance in such pastoral areas are difficult because human populations are relatively small and highly mobile, and they move their livestock across large areas with few roads and means of modern communications [23,24]. In such situations, conventional approaches to veterinary research and disease surveillance require considerable flexibility and commitment. Given the resource and logistical constraints in such pastoral areas, pastoralists themselves are a valuable source of disease information [25]. Cattle managed under pastoral extensive system are persistently at risk of contracting contagious diseases, including RVF, due to continuous mixing of herds with vectors at grazing and watering points, and as well as social exchange practices (socio-cultural practices of giving out cattle as dowry and gifts) [22,26,27], and these practices could inhibit RVF and other livestock diseases control strategies.

Rift Valley fever epidemiology in nomadic pastoral herds is poorly understood in Nigeria due to paucity of research and surveillance information on the disease. Also, no clinical case of the disease has been formally reported in livestock in Nigeria [28]. Besides, past studies suggested that RVFV circulated among sedentary ruminants and humans in Nigeria [29,30]. Currently, no information is readily available on the nomadic pastoralists’ knowledge about the disease in livestock in the country. Availability of such science-based information in nomadic pastoral communities is needed to better understand RVF epidemiology for adequate development of surveillance and control strategies for the disease in the nomadic pastoral settings. The objectives of this study were: (1) to assess perceived relative burden and seasonality of RVF in nomadic cattle herds and validate the burden with sero-prevalence impact; and (2) assess perceived risk factors for the disease and risk pathways for RVFV in nomadic pastoral cattle herds of North-central Nigeria. We hypothesized that nomadic pastoralists do not possessed significant existing veterinary knowledge and traditional oral history about RVF and other cattle diseases and, therefore, they cannot be used for epidemiological investigation of the disease for surveillance and research.

## Materials and methods

### Ethics statement

Research protocol was approved by the Niger State Ministry of Livestock and Fisheries Development, Minna Research Ethics Committee (ref, MLFD/NGS/677). Participants were provided with verbal information on the objectives of the study. Informed consents of respondents were verbally obtained before commencement of each section of participatory exercise in every community and none declined participation. They were assured of voluntary participation, confidentiality of responses and the opportunity to withdraw at any time without prejudice in line with the Helsinki Declaration [35]. Verbal information and informed consent were deemed necessary due to very low literacy levels among pastoralists.

### Study area

**Fig 1:**
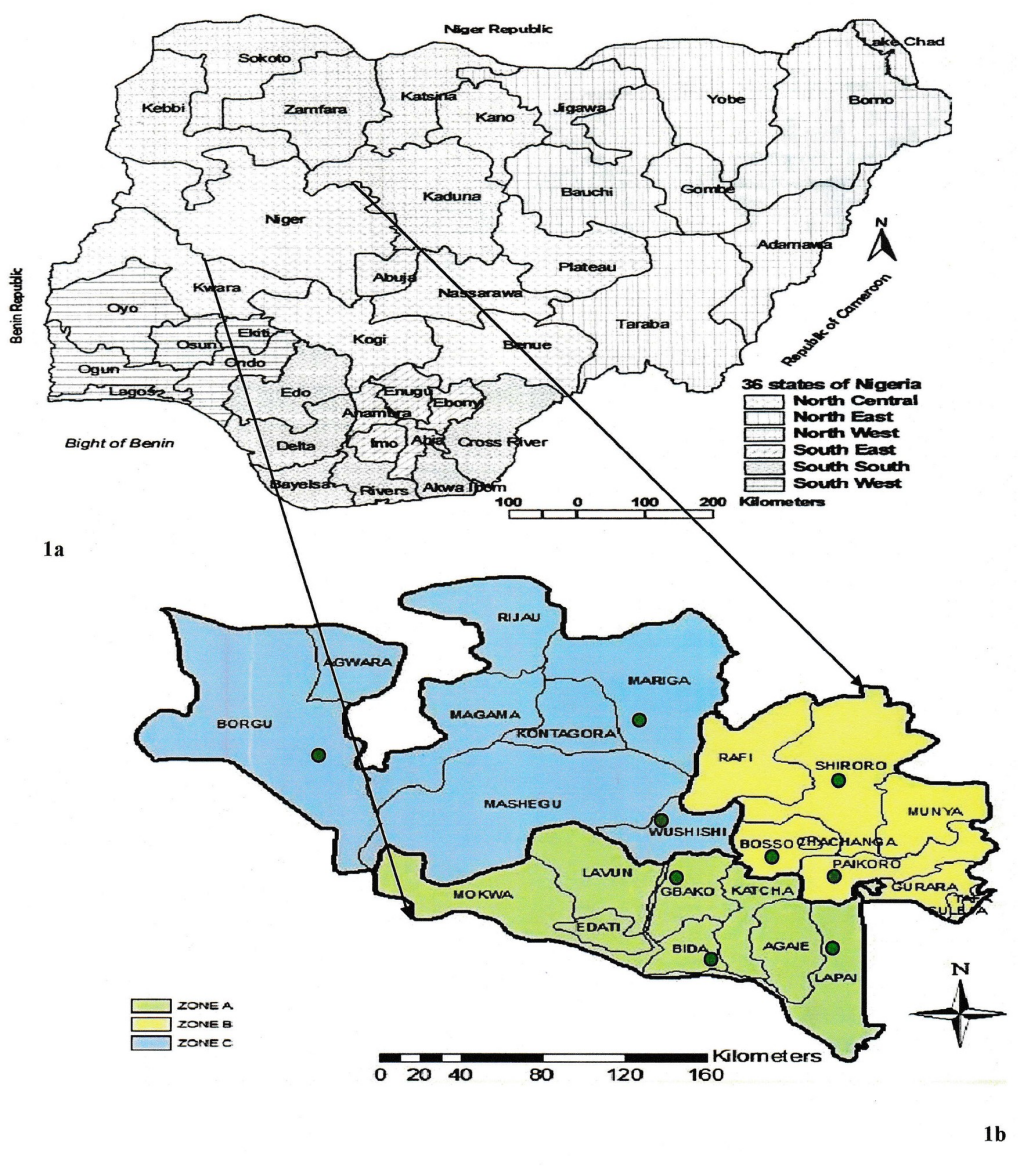
Study area. 1a: Map of Nigeria showing the location of Niger State. 1b: Map of Niger State showing the three Agro-geographical zones A, B and C with Fulani nomadic pastoral communities’ PE locations (in green circles).

The study was conducted in Niger State, located at the Southern Guinea savannah in the North-Central geopolitical zone of Nigeria, between latitude 8° 20′ N and 11° 30′ N, and longitude 3° 30′ E and 7° 20′ E. It is one of the 36 states of Nigeria and provides transit routes for pastoral nomads on seasonal migrations from the northern parts to the southern parts. The state has three Agro-geographical zones (Fig 1), with variable climatic conditions. These are: Agro-geographical zone A (Southern zone), with eight local governments areas (LGAs), many rivers, streams and ponds, fadamas for rice farming, Jebba hydro-electric dam and large grazing lands; Agro-geographical zone B (Eastern zone), with nine LGAs, many mountains, trees, and rivers and streams, Shiroro hydro-electric dam, arable and large grazing lands; and Agro-geographical zone C (Northern zone), with eight LGAs, few rivers and streams, Kainji hydro-electric dam, Kainji National Game Reserve, arable and large grazing areas, and many stock routes. Also, has an international border with the Republic of Benin at its western border, which is porous [31]. The coordinates of Fulani nomadic pastoral communities’ PE locations (in green circles) in the Agro-geographical zones are: Lapai (GPS coordinate: N09.0102°, E006.61729°); Eyagi (N09.13506°, E006.00618°); Lemu (N09.17155°, E006.01972°); Paiko (N09.43533°, E006.60745°); Kuta (N09.84643°, E006.71782°); Bosso (N09.66275°, E006.47691°); Wushishi (N09.69760°, E006.05682°); Bobi grazing reserve (N09.16715°, E005.91701°); and Borgu (N09.91455°, E004.33400°). The electronic maps were obtained from the Niger State Geographical Information System Office in Minna, Nigeria. The agro-geographical zones were based on the exiting demarcations of the state into three agricultural areas. The GIS software program, ArcGIS 9.3 (Environmental Systems Research Institute (ESRI), Inc., Redlands, USA) was used for plotting the PE sites (Fig 1).

Niger State experiences four distinct seasons: early rainy season that spans between April and June; late rainy season that spans between July and September; early dry season from October to December; and late dry season that spans from January to March. It has mean annual rainfall of about 150 cm and duration of about 180 days. The state has average relative humidity of 58.6% and means lowest and highest temperature of 22 °C and 39 °C, respectively. It has an estimated cattle population of 2.4 million cattle, which are mostly in the custodies of pastoralists [31].

### Study design and social structure of target populations

Participatory Epidemiology (PE) survey was conducted using participatory approaches and methods in nomadic pastoral communities domiciled within the three Agro-geographical zones of the state. Also, a cross-sectional investigation was carried out in nomadic pastoral herds to validate outcomes of relative burden from PE. Both were used to collect qualitative, semi-quantitative, and quantitative data from the pastoralists between January and December 2015.

The target populations were the Fulani nomadic pastoralists, who are seasonally mobile, with scattered herds of local breeds of cattle (Bunaji, Rahaji and Bokoloji), domiciled in remote areas of the state during the study period. The average number of herds that formed a nomadic pastoral community was 28, each managed by herd head or owner (a man, his wives and children, or an elderly widow and her children). The average number of animals in a herd was 65 cattle of variable ages and sex.

### Definitions

In this study, nomadic pastoral herd was defined as cattle herd in Fulani ethno-cultural group that keeps mainly cattle, usually large herd of 60 cattle and above, and takes part in year-round long movements on large range for grazing and in search for water, without permanent homestead.

Participatory epidemiology is the systematic use of participatory approaches and methods to improve understanding of RVF and other cattle diseases in the Fulani nomadic pastoral communities. PE is an emerging field that is based on the use of participatory techniques for systemic harvesting of epidemiological intelligence information contained within community observations, existing veterinary knowledge (clinical and epidemiological information of diseases) and traditional oral history to improve understanding of diseases and options for animal disease [25,32].

Relative burden was the pastoralists’ perceived effects of RVF among other important cattle diseases, characterized by such indicators as abortion in pregnant cows, deaths of neonatal calves and production losses (milk and weight losses).

The Risk pathway analysis is systematic assessment of the paths along which a disease could enter into a susceptible animal population, spread and establish, and consequences of its occurrence in such population. It is an integral to risk assessment estimation, in semi-quantitative term, for likelihood of spread of a disease through identified pathway and consequence of such spread [33].

### Sample size and sampling procedure

In the PE investigation, nine Fulani nomadic pastoral communities were purposively selected across the state, three in each agro-geographical zone, in a manner that allowed for adequate spread in each zone. They were Lapai, Eyagi, Lemu, Paiko, Kuta, Bosso, Wushishi, Bobi grazing reserve and Borgu nomadic pastoral communities. Key criteria for selection of the communities included: nomadic herding of cattle as main source of livelihood, and remoteness of the settlements. Also, three pastoral key informants were purposively selected in each community to organize and lead other pastoralists for action-oriented participatory exercises. However, for each pastoral community the number of other willing participants that participated was not restricted. A total of 27 key informants were purposively selected for the study.

For the sero-prevalence study, sample size was computed for finite population correction factor N using the Open Source Epidemiologic Statistics for Public Health **(**OpenEpi) 2.3.1 software [33]**,** with power set at 50%, and 10% margin of error at 95% confidence level. A sample size of 97 nomadic cattle was computed. A two-stage sampling method was conducted to select the animals. In the first stage, 15 nomadic herds were purposively selected across the state, five in each Agro-geographical zone. Each herd had an average of 65 cattle. In the second stage, at least five cattle were randomly selected from each herd. Systematic random sampling method was used to select animals from herds. Sampling interval of 13 was used, obtained from dividing average number of cattle in a herd (n = 65) by number of animals expected to be sampled in it (n = 5).

### Participatory data collection

Advocacy visits were made to each community a week prior to the proposed participatory exercises and necessary permission obtained from *Dikkos* (Fulani community leaders). Key informants were told about the survey as being meant to investigate their perceived burdens of important cattle diseases in nomadic pastoral communities, in relation to the effects on stocks’ productive performances, using their existing knowledge and traditional oral history about cattle diseases, and outcomes were expected to be used for designing control strategies. However, Rift Valley fever was not specifically mentioned to avoid bias, especially where it is not a major challenge. PE was conducted by an appraisal team trained on participatory methods as described [36,37], and participatory rural appraisal tools of semi-structured interview, proportional piling, matrix scoring, seasonal calendar and triangulation were used. Qualitative (identified diseases/conditions and factors) and semi-quantitative (piling and scoring exercises) data were collected by used of these PE techniques as previously described [38].

### Key informants and semi-structured interview

Key informants were the traditional Fulani pastoralist leaders or elders in the communities who command much respect from their members. According to the Fulani tradition, they are considered to be more knowledgeable than others on animal health and production management. They led other pastoralists in their respective communities to group participatory exercises. Semi-structured interview **(**SSI) began with introduction of the appraisal team and explanation of purpose of the visit. During each session of SSI in the communities, which ran for about three hours, general information about cattle diseases encountered and their perceived predisposing risk factors was discussed. In order to facilitate discussion, the appraisal team asked questions that began with more general topics on cattle management followed by specific important diseases affecting their cattle. These were guided by a pre-tested checklist of open-ended questions that standardized discussions, and questions were probed depending on the key informants’ responses. Mentioned diseases were probed and expanded descriptions of their clinical and epidemiological manifestations obtained. Also, participants were told to identify and list perceived risk factors for various diseases mentioned and were asked to explain how the factors could predisposed to occurrence of each listed disease. Local languages of *Hausa* and *Fulfulde* were used to conduct the interviews and detailed descriptions of RVF and other cattle diseases in each pastoral community were collected, translated to English and recorded.

### Proportional piling of most important cattle diseases

Materials used in this exercise were counters (pebbles), flipcharts and permanent markers. In each community, pastoralists were asked to give a list of ten most important diseases perceived to be affecting their cattle within a ten-year period preceding time of interview. Pastoralists often used local disease names to identified cattle diseases. When they provided syndromes rather than specific names of diseases, probing was carried out using open-ended questions to characterize the syndrome whilst trying not to guide them. Names of the diseases and descriptions given by pastoralists were later validated at the Zonal Veterinary Offices and by the veterinary field epidemiology experts’ opinions. Once the participating pastoralists and appraisal team had compiled list of diseases, ten circles were drawn on flip charts, with each representing a mentioned disease. Pastoralists were given 100 pebbles and instructed to pile them in the circles proportionally to the perceived impact of each disease to their herds, in terms of loss in milk and weight loss, to mention few. The appraisal team then counted pebbles placed in each circle to give a proportion that represented relative burden of each disease in that pastoral community.

### Matrix scoring for clinical manifestations of most important cattle diseases

Informal interview with pastoralists was conducted by the appraisal team to identify six most important cattle diseases among those mentioned during proportional piling technique and were used for matrix scoring technique [24,37]. Matrix scoring of the perceived clinical signs associated with RVF and other five important cattle diseases was conducted. Pastoralists were asked to list fifteen (15) important clinical indicators that are associated with the six identified diseases. Listed clinical indicators were then placed on *y*-axis of drawn matrix chart on the flip charts, while the six diseases placed on *x*-axis. Ten (10) pebbles were allocated to each clinical sign (indicator) and participants were asked to divide the pebbles proportionally to the perceived importance of each indicator to a disease in the matrix. Appraisal team then counted the pebbles in each box to give a proportion that represented relative association of a clinical sign with a disease.

### Proportional piling of perceived risk factors for RVF occurrence

Participants in each pastoral community were asked to list ten most important perceived risk factors that could predisposed to RVF occurrence in cattle herds. Once consensus was reach on the factors, the appraisal team probed each factor’s epidemiological significance to RVF occurrence in cattle herds. Ten circles were drawn on flip charts and each represented a mentioned risk factor. Pastoralists were given 100 pebbles and asked to pile them in the circles proportionally to the perceived influence of each factor on RVF occurrence in the herds. The appraisal team then counted the pebbles in each circle to give a proportion that represented influence of the factors on diseases’ occurrence in each nomadic pastoral community.

### Matrix scoring for RVF risk pathways analysis

Since RVF was identified during SSI as one of the ten most important cattle diseases in their communities, key informants were further asked to identify factors (indicators) that could influence entry of RVF causative agent (RVFV) into cattle herds, spread within herds and consequences or effects in herds. Pathways matrices were drawn on flip charts for the risk pathways (entry, spread and consequences) of the disease in herds. Participants were further probed on influence of each factor (indicator) on the three pathways. The identified epidemiological factors (indicators) were placed on *y*-axis while the three pathways formed *x*-axis. Fifteen (15) pebbles were allocated to each factor and participants asked to score them proportionally to the perceived influence of an indicator on RVFV in each pathway. Appraisal team then counted the pebbles in each matrix to give a proportion that represented relative influence of a factor (indicator) on RVFV in a pathway.

### Seasonal calendar of RVF occurrence

Seasonal calendar exercise on occurrence of RVF and other five most important cattle diseases was carried out. A seasonal calendar of 12 months (January to December) mentioned by pastoralists was considered. A matrix calendar of 12 months was designed on flip charts and were further grouped into four based on four sub-seasons identified by Fulani pastoralists for occurrence of the cattle diseases in the state, namely: *Kaka* or early dry season (October to December), *Rani* or late dry season (January to March), *Bazara* or early rainy season (April to June), and *Damina* or late rainy season (July to September). Seasons on the matrices were presented on *x*-axis, while diseases (indicators) were placed on *y*-axis. Ten (10) pebbles were allocated to each disease (indicator) and participants told to score pebbles proportional to the perceived relative weight of each disease’s occurrence in each season. Appraisal team then counted the pebbles in each matrix to give a proportion that represented the relative weights of RVF occurrence in each season.

### Triangulation and key biological sampling

Data obtained from participatory exercises in each community were cross-checked and further debated among participants until a consensus view or agreement was arrived at. The studied nine pastoral communities’ results were also triangulated (compared) at the end of all PE exercises by the appraisal team. Results were analyzed and mean outcomes of the perceived relative burdens and seasonal trends of RVF and some important cattle diseases obtained. Also, appraisal team triangulated the results of the epidemiological predisposing factors and risk pathways from the nine communities to obtain their averages. The final semi-quantitative (participatory) results obtained from the nomadic participants were further cross-checked with the Area veterinary officers in the state and field veterinary epidemiologists for final validities.

Key biological sampling was the final method of triangulation in PE [38]. The participatory (qualitative and semi-quantitative) data obtained from PE exercises were finally validated by results from collected and analyzed ‘serum’ samples in the form of sero-prevalence (quantitative).

### Serum samples collection and enzyme immunoassay

Ten (10) ml of whole blood was taken from jugular vein of each selected cattle with a sterile 10 ml syringe and 18 × 1½” gauge needle for each cattle. These were immediately placed into an ice bath slant and transported to the laboratory within seven hours. The clot was allowed to form in syringe in the field, which was later transferred into plastic tubes and centrifuged at 3000 rpm for 20 min, decanted and stored at −20 °C until analyzed.

A competitive Enzyme Linked Immunosorbent Assay (c-ELISA) was used. An indirect competitive ELISA (cELISA) kit (ID Screen Rift Valley Fever Competition multispecies ELISA; IDVet Innovative Diagnostics, Grabels, France) was used according to the manufacturer’s instructions for detecting RVFV-specific antibodies. The kit has a validated diagnostic sensitivity and specificity of 100% [39]. The plates were read using ELISA plate reader at 450 nm. Results were interpreted by expressing as percentage of the positive serum (PP) using the formula: (mean OD of duplicate test serum) / (mean OD of positive control) × 100; where positive and negative cut-off values are determined by receiver operating characteristic (ROC) curve analysis. The cut-off values used were: PP values (%): negative <4; suspicious 4–6; and positive>7.

### Data management and analysis

Data obtained from each PE exercise were recorded in a field note-book and results of exercises that created visual representations were captured on a digital camera. Data obtained were qualitative and semi-quantitative in nature; the former were probed and discussed during SSI without being subjected to formal statistical analyses, while the later (mostly from piling, scoring and ranking exercises) were summarized and entered into a Microsoft Excel 7 database (Microsoft Corporation, Redmond WA, USA) and stored. The Kendall’s Coefficient of Concordance W statistic, a non-parametric statistics [40,41,42] was used to assess levels of agreements among the key informants and other participants at 95% confidence level. Descriptive statistics of rate and mean were used to describe the relative burden and seasonal occurrence of RVF among other cattle diseases from proportional piles and seasonal calendars. Kendall’s coefficient of concordance was also used to understand degree of agreement among the key informants on perceived risk factors and clinical manifestations of RVF in cattle herds within the study area. P<0.05 indicates statistical significance of the agreements.

The risk pathway analysis is based on three possible stages; assessment (Entry, Exposure/spread and Consequence), communication and management. It involved estimation of probabilities of RVF occurrence considering its epidemiology [43]. However, we used pathway by assessment and communication because our target was to grade consequences status of the factors and communicate feedback to the stakeholders. The consequences of factors in risk pathways were assessed thus: low risk (L) for piles that ranged from 1 to 5; medium risk (M) for piles that ranged from 6 to 10; and high risk (H) for piles that ranged from 11 to 15.

Descriptive statistics were used to describe obtained serological data. In the descriptive analysis, frequency and proportion were used and analyzed using the Open Source Epidemiologic Statistics for Public Health (OpenEpi) version 2.3.1 [34] at 95% confidence level.

## Results

### Proportional pile for RVF relative burden

**Fig 2:**
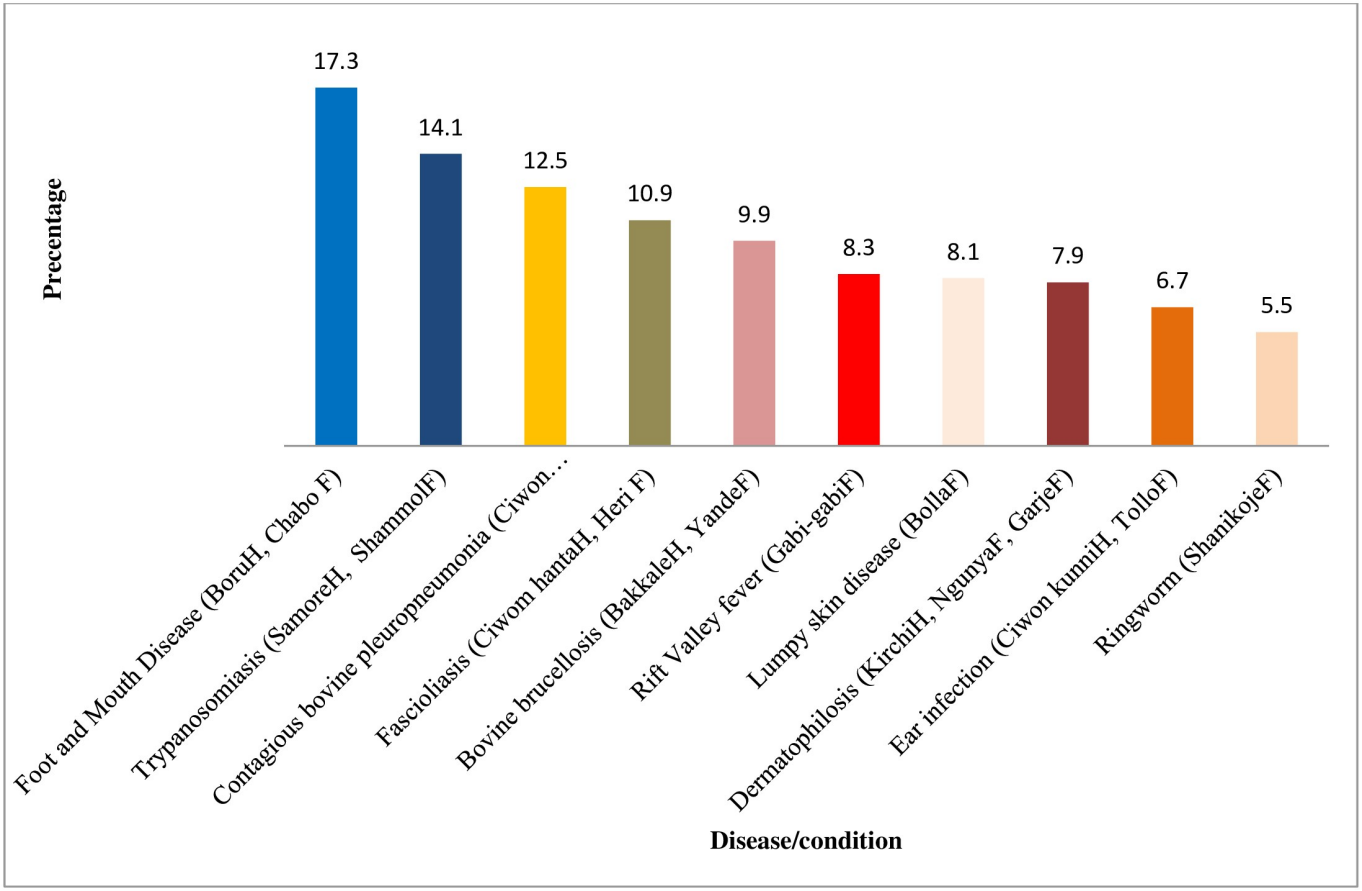
Mean proportional piles (rank order) of relative burden of RVF and some cattle diseases in nomadic pastoral communities of Niger State. Numbers in parenthesis are the average proportions of each disease. Superscripts *H* and *F* are the local names in *Hausa* and *Fulfulde*, respectively.

Key informants and other pastoralists demonstrated detailed existing veterinary knowledge and traditional oral history of cattle diseases during the participatory exercises in their communities, with RVF been cited as one of the most important cattle diseases. Although the range of cattle diseases that pastoralists mentioned varied somewhat between the nine nomadic pastoral communities under survey, RVF featured prominently in all communities. However, cattle diseases that were averagely ranked with relative high burdens in relations to effects on cattle productive performances (production and reproduction) were Foot-and-mouth disease (*Boru*^*H*^, *Chabo*^*F*^), Trypanosomosis (*Samore*^*H*^, *Shammol*)^F^, Contagious bovine pleuropneumonia (*Ciwon huhu*^*H*^, *Huttu*^*F*^), Fasciolosis (*Ciwom hanta*^*H*^, *Heri*^*F*^), Bovine brucellosis (*Bakkale*^*H*^, *Yande*^*F*^), Rift Valley fever (*Gabi-gabi*^*F*^), Dermatophilosis (*Kirchi*^*H*^, *Ngunya*^*F*^, *Garje*^*F*^), Lumpy skin disease (*Bolla*^*F*^), among others (Fig 2). The mean proportional piles (relative burden) of RVF among other cattle diseases in Lapai, Lemu, Eyagi, Paiko, Kuta, Bosso, Wushishi, Bobi Grazing Reserve, and Borgu Fulani nomadic pastoralist communities was 8.3%, which was judged by pastoralists to be sixth most important cattle disease in terms of impacts on cattle. Pastoralists’ agreement on the relative burdens of these diseases in Niger State was strong (W = 0.6855) and statistically significant (p<0.001).

### Matrix scores for clinical manifestations of RVF and some cattle diseases

The consistent six most important cattle diseases mentioned by pastoralists in all the nine communities were Foot-and-mouth disease (*Boru*^*H*^, *Chabo*^*F*^), Trypanosomosis (*Samore*^*H*^, *Shammol*)^F^, Contagious bovine pleuropneumonia (*Ciwon huhu*^*H*^, *Huttu*^*F*^), Fasciolosis (*Ciwom hanta*^*H*^, *Heri*^*F*^), Bovine brucellosis (*Bakkale*^*H*^, *Yande*^*F*^), and Rift Valley fever (*Gabi-gabi*^*F*^). Nomadic pastoralists proved to be knowledgeable at recognizing clinical signs of RVF in their cattle herds. Mean matrix scores of clinical signs associated with the six major diseases, including RVF, are presented in Table 1. Fulani nomadic pastoralists in the studied communities called Rift Valley fever *Gabi-gabi*, denoting a disease associated with high fever, diarrhea and sudden death in newborns (calves) as well as abortions in pregnant cows. Mean matrix scores of prominent clinical signs that were consistently scored to be associated with RVF across the nine nomadic communities were fever (3.1), anorexia (2.1), abortion (4.1), nasal discharge (3.3), neurological disorder (8.4), diarrhoea (3.2), and sudden death (4.4). There was strong agreement among the key informants on the clinical manifestations of RVF and other mentioned cattle diseases (W = 0.6687) and were statistically significant (p<0.001). Words in parenthesis are the *Hausa* or *Fulfulde* names.

**Table 1:** Mean matrix scores of fifteen listed clinical signs associated with Rift Valley fever and some important cattle diseases in pastoral communities of Niger State.

Disease						
Clinical sign	Trypanosomiasis %	FMD %	CBPP %	Fascioliasis %	Brucellosis %	RVF%
Fever	0.3	2.6	1.7	0.2	2.1	3.1
Salivation	1.1	5.9	1.6	0.4	0.0	0.3
Difficult breathing	0.0	0.0	8.1	0.0	0.0	0.6
Mouth lesions	0.0	9.5	0.0	0.0	0.0	0.0
Feet lesions	0.0	9.8	0.0	0.0	0.2	0.0
Anorexia	1.6	3.9	1.7	0.5	0.0	2.1
Emaciation	3.8	5.1	1.9	3.3	1.1	0.6
Abortion	1.8	0.0	0.0	0.0	4.4	4.1
Swollen knee joints	0.0	0.0	3.1	0.0	6.8	0.0
Cough	2.7	0.0	4.6	1.2	0.0	0.7
Nasal discharge	1.4	3.1	2.1	1.7	0.0	3.3
Neurological disorder	0.0	0.5	0.7	0.0	0.3	8.4
Lacrimation	7.9	0.0	0.6	1.3	0.0	1.3
Diarrhoea	1.6	0.3	0.0	5.4	0.0	3.2
Sudden death	1.8	2.3	3.4	0.0	0.2	4.4

### Proportional piles of risk factors associated with RVF

**Fig 3:**
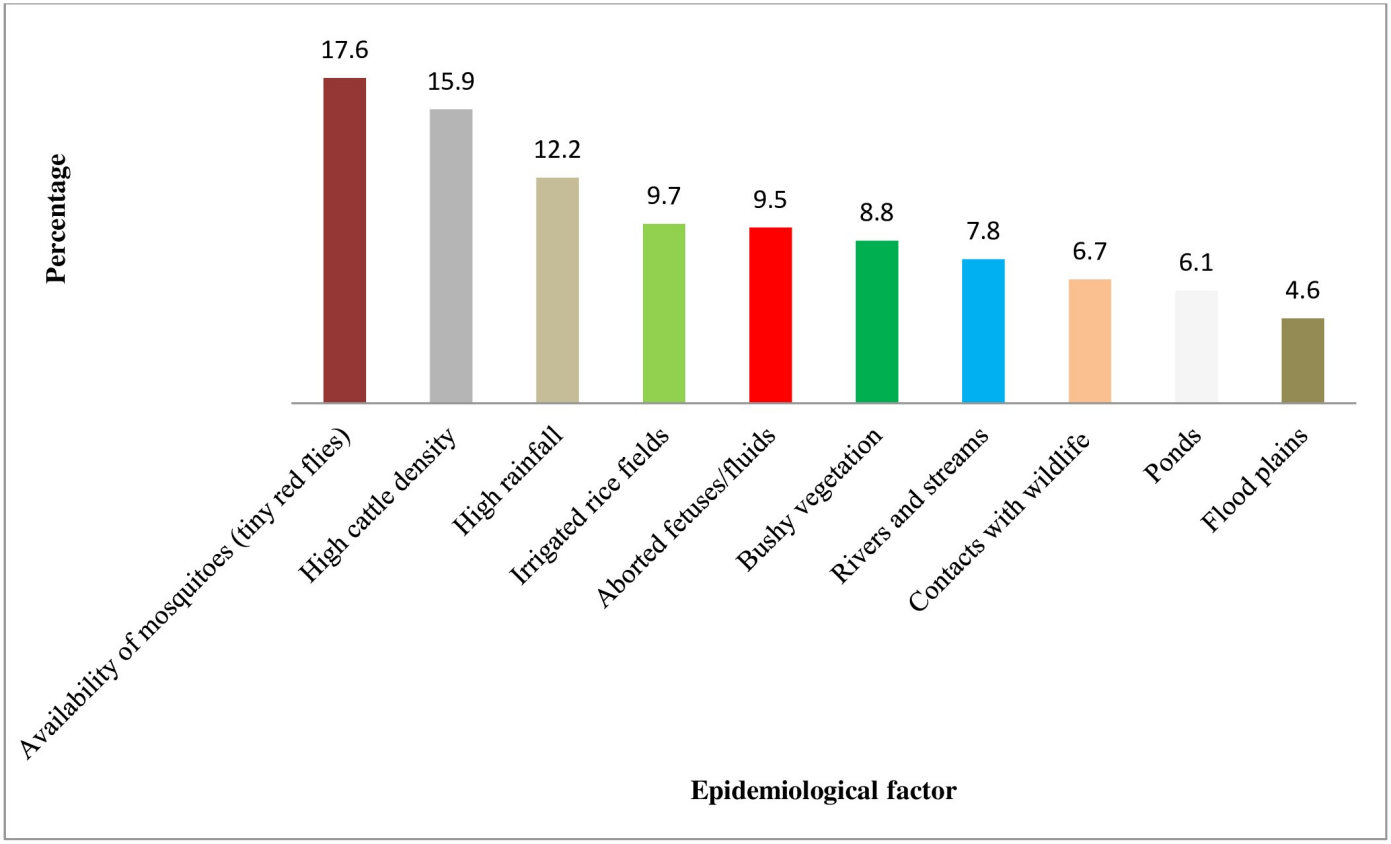
Mean proportional piles of the perceived risk factors predisposing to Rift Valley fever occurrence in cattle herds in nomadic pastoral communities of Niger State. Numbers in parenthesis are the average proportions of each risk factor.

The mean proportional piles of pastoralists’ perceived risk factors identified to influenced occurrence of RVF in their communities were: availability of mosquitoes (tiny biting flies) (18 piles, 17.6%), high cattle density (16 piles, 15.9%) and high rainfall (12 piles, 12.2%). Other risk factors piled were irrigated rice fields (10 piles, 9.7%), aborted foetuses and fluids (10 piles, 9.5%), bushy vegetation (9 piles, 8.8%), rivers and streams (8 piles, 7.8%). Also, ponds (6 piles, 6.1%), contacts with wildlife (7 piles, 6.7%), flood plains (5% piles, 4.6%) were mentioned as risk factors for the disease in their communities (Fig 3). Pastoralists’ agreement on the risk factors was strong (W = 0.8372) and statistically significant (p<0.001).

### Matrix scores of RVF virus risk pathways

Mean matrix scores for the Entry pathway of RVF agent into the nomadic pastoral herds were: presence of RVFV infected mosquitoes (tiny biting flies) (7.9), presence of infected cattle in herds (8.4), and contacts of herd with infected wild animals at grazing (10.1). The mean matrix scores for the Spread pathway of RVFV in the herds were bites of infected mosquitoes at the grazing and watering points (5.1), contacts with infected aborted foetuses and fluids at the herd settlements and grazing areas (7.8), and contaminated pasture with aborted fetuses and fluids (9.7). Spread of RVFV through mosquitoes’ bites was perceived to be the most possible form of spread with the mean matrix scores consequence high risk of 9.9, while contaminated pasture with infected aborted fetuses and fluids had low risks with 2.6 mean matrix scores (Table 2). Nomadic pastoralists’ agreement on risk pathways was strong (W = 0.6922) and statistically significant (p<0.001).

**Table 2:** Mean matrix scores of risk pathways for entry, spread and consequences of RVF and associated epidemiological factors in cattle herds of nomadic pastoral communities in Niger State.

Pathways			
Epidemiological factor	% Entry into herd	% Exposure/spread in the herd	% Consequence
Presence of infected mosquitoes (tiny red flies)	7.9	1.7	5.1
Presence of infected cattle in herds	8.4	1.3	4.8
Contacts of herds with infected wild animals at grazing	10.1	2.4	2.7
Bites of infected mosquitoes at grazing and watering points	0.3	5.1	9.9
Contacts with infected aborted fetuses/fluids at herd sites	1.3	7.8	6.3
Contaminated pasture with aborted infected foetuses	1.5	9.7	2.6

### Seasonal occurrence of RVF

**Fig 4:**
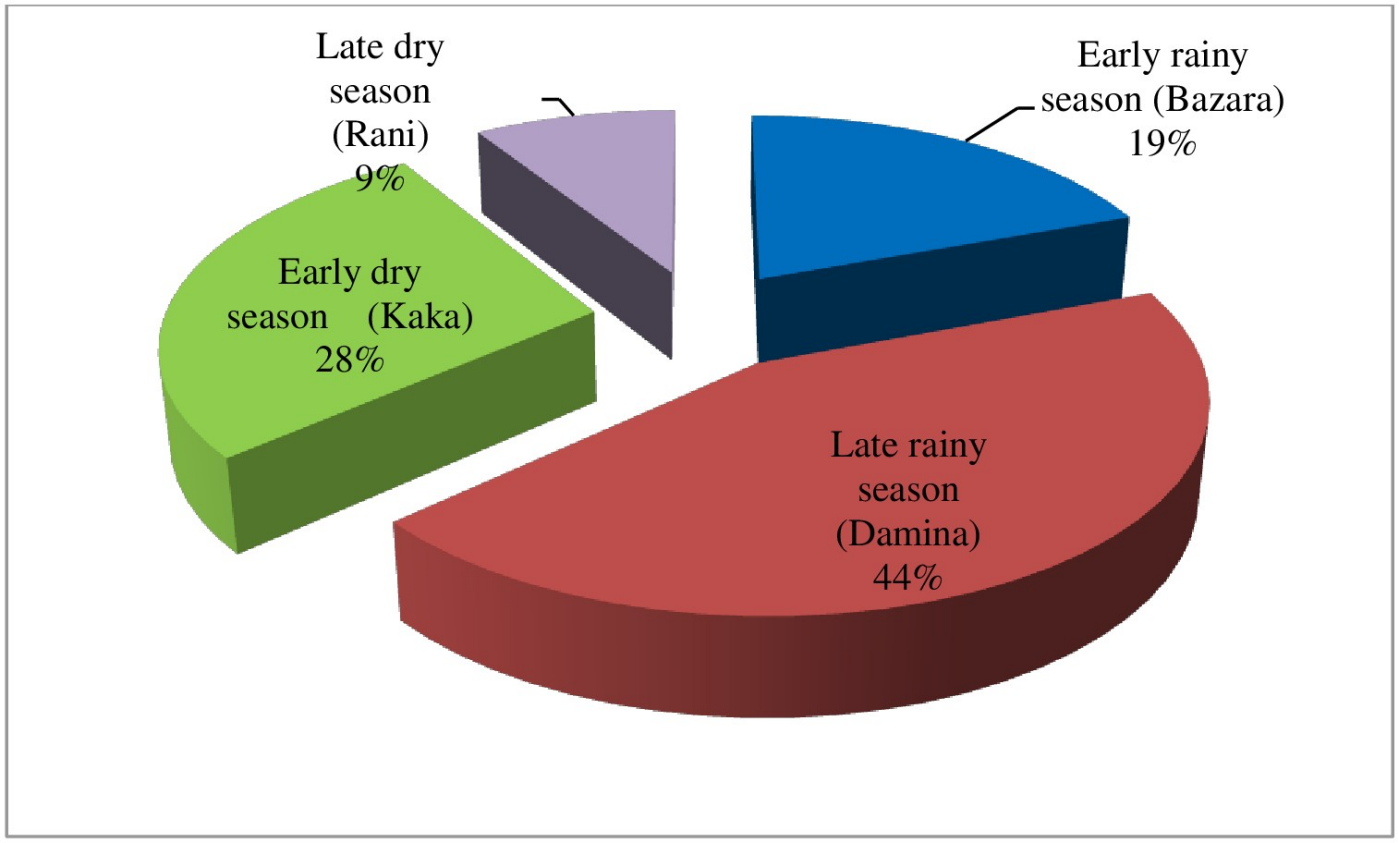
Proportions of mean seasonal scores of Rift Valley fever in nomadic pastoral communities of Niger State. Words in parenthesis are the local names for the seasons.

Although Rift Valley fever was mentioned to occurred in all seasons as indicated in the seasonal matrix calendar, the disease was however more prevalent in one season than another. Key informants and other pastoralists scored RVF to occur more in *Damina* or late rainy season (5.3), followed by *Kaka* or early dry season (3.3) (Fig 4). Pastoralists’ agreement on seasonal occurrence of RVF among some cattle diseases was strong (W = 0.8719) and statistically significant (P<0.001).

### Sero-prevalence of RVF

Of the 97 cattle sampled and their sera examined for antibodies to RVFV antigen in nomadic herds using cELISA assay, 11.3% (11/97; 95% CI: 6.1, 18.9) were sero-positive (Table 3). In the three Agro-geographical zones, highest sero-prevalence of 18.2% (95% CI: 7.7, 34.0) was observed in Agro-ecological zone B, while lowest of 3.1% (95% CI: 0.2, 14.5) was observed in Agro-ecological zone C (Table 3).

**Table 3:** Cattle-level sero-prevalence of RVF in nomadic cattle herds of Niger State, North-Central Nigeria.

Agro-geographical zone	Number of cattle sample	Number of cattle positive	Percentage positive	95% Confidence interval
A (Southern)	32	4	12.5	4.1, 27.5
B (Eastern)	33	6	18.2	7.7, 34.0
C (Northern)	32	1	3.1	0.2, 14.5
Total	97	11	11.3	6.1, 18.9

## Discussion

To our knowledge, this study was the first to investigate Rift Valley fever occurrence using Participatory Epidemiology techniques, validated quantitatively with sero-prevalence at same survey, in Fulani nomadic pastoral communities. The nomadic pastoralists provided more detailed and accurate clinical descriptions of important diseases affecting their cattle, including RVF. Pastoralists called RVF *Gabi-gabi*, denoting disease associated with high fever, diarrhoea, lacrimation, nasal discharge, neurological disorder, abortions in pregnant cows sand sudden death in newborns (calves). This suggests that the PE approaches can be used to detect RVF occurrences earlier by taking advantage of pastoralists’ observations through integration of active syndromic surveillance, such as participatory disease surveillance (PDS) geared to the level of outbreak probability [38,44]. Although RVF was not the most prevalent disease that affects cattle in the studied Fulani nomadic communities, it proves to be one of the important cattle diseases that have greatest impacts on their socio-economic livelihoods. The variable clinical manifestations reported by the pastoralists in different communities may be caused by differences in the local ecology of the RVF virus or its virulence. It is known that multiple RVF viral lineages existed [45].

Through proportional piling, pastoralists identified the availability of mosquitoes, high cattle density and high rainfall, irrigated rice fields, and aborted fetuses/fluids as leading risk factors predisposing to RVF occurrence in this study. These findings are consistent with reports that showed strong association between severe infections of RVF and large numbers of animals and mosquitoes in the 2006/2007 RVF outbreaks in Kenya [46]. High cattle density as an important factor for RVF occurrence should be expected in Nigeria, since ruminants are the main animal host of RVFV [47]. It has been observed in many studies that RVF outbreaks and endemicity are closely linked to heavy rainfall and high numbers of mosquitoes because high rainfall drives mosquito emergence [48,49,50,51]. Presence of agricultural irrigation in grazing areas was observed to be associated with risk of RVF occurrence in this survey. This is understandable as irrigation is known to directly benefit mosquitoes by increasing their habitat availability for larvae [52]. Nomadic pastoralists have the habit of tethering their animals by their huts at night for fear of theft, providing an animal reservoir of RVFV in proximity of humans, which have been reported to increase the number of *Culex* mosquitoes in the communities that practice this culture [53]. Pastoralists’ pilings on wildlife as factor predisposing for RVF occurrence is in consonant with findings of a study that detected RVF antibodies in warthogs, gerenuk, waterbuck and buffalo [54].

Other described risk factors for RVF occurrence in cattle herds observed in this survey, such as dense vegetation and proximity to perennial water bodies (rivers and streams) as well as ponds, have been previously reported to predisposed to RVF outbreaks, especially in Senegal [55;56,57]. In our study area, the low-lying areas close to Rivers Niger and Kaduna, as well as the three hydro-electric dams, are subjected to regular flooding during rainy seasons. This phenomenon provides abundant mosquito breeding sites in low-lying areas. Also, frequent water logging in the Southern agro-geographical area of the state has lead to large areas being used for wetland rice cultivation. A report from the 2006/2007 RVF outbreaks in Kenya found areas with soils that retain water to be more affected than in other areas [58]. In this study, matrix scoring results showed that the nomadic pastoralists were aware of heavy rains and flooding nature in the grazing areas, which can influence occurrence of RVF in their herds.

Three pathways for risk assessment (Entry, Exposure and Consequence) were used in this study, in relation to the possible entry, transmission and spread of the of the RVF virus into herds. The observed entry risk pathways for RVF in nomadic pastoral herds were through infected mosquitoes, infected domestic animals, and infected wild animals. These findings were corroborated by reports of an investigation on the scientific pathways toward public health prevention and response to RVF [59]. The identified exposure/spread risk pathway associated with infected mosquitoes’ bites at cattle grazing and watering points, contacts with infected aborted fetuses/fluids at grazing areas and herd sites, and herds’ exposures to pastures and environments contaminated by infected aborted foetuses and fluids in this study were indications of nomadic pastoralists’ understanding of risk pathways for RVF occurrence. Noteworthy is the fact that pastoralists considered contacts with infected aborted foetuses as a pathway for exposure and spread of RVFV in herds, though of low risk level. This is consistent with reports of a previous survey that found aborted foetuses to be a single most important factor directly associated with exposure and spread of RVFV [46]. We also observed highest risk pathway to be bites of infected mosquitoes and lowest to be through exposure to contaminated environment with infected fetuses and fluids.

We observed occurrence of RVF and other cattle diseases to be associated with distinct seasonal patterns in nomadic pastoral communities. Across the four seasonal periods, there were marked differences in numbers of RVF scores recorded. It was also observed that RVF risk has distinct seasonal patterns. Pastoralists mentioned RVF occurrence to be at its highest during late rainy season, which they named *Damina*, and lowest during the late dry season, called *Rani*. Late rainy season could favour fast breeding of RVFV carrying mosquito species, and hence with seasonal abundance than other seasons. In contrast, it has been reported that, although there were marked differences in RVF occurrence across seasonal periods, it was more during the first and second quarters of the year (January–June) [60]. However, RVF virus activities often occur across the year, but more associated with seasons of rainfall during non-epidemic periods [61].

Pastoralists’ existing veterinary knowledge about RVF among other cattle diseases in this study was significant, as they were able to collectively name and describe clinical manifestations of cattle diseases and syndromes in remote rural areas at low cost. The resultant finding in the present study was the pastoralists’ ability to have identified major cattle diseases, including RVF, with their epidemiological parameters in the target communities. These can serve as potential active surveillance for the diseases and strategic process for control purposes. Therefore, with important roles played pastoralists in the PE exercises, they can serve as a fulcrum for surveillance of livestock diseases in remote settlements in Nigeria, as identified in previous studies on livestock diseases and management [62,63].

The qualitative relative RVF burden of 8.3% at PE approach was validated by the RVFV sero-positivity of 11.3%. Both were not for comparison but confirmation of RVF occurrence in North-central Nigeria. They were validation findings, though of different values and techniques. The overall cattle-level sero-prevalence indicates that there was a considerable level of cattle challenged with RVFV infections without manifesting clinical signs, but with evidence of detectable antibodies. It is possible to conclude that RVFV is enzootic in North-central Nigeria because the presence of IgG antibodies in cattle. This is consistent with a part of reports on the previous national survey [29]. Noteworthy, the RVFV-specific antibodies detected in the cattle indicated natural infection as RVF vaccination has never been practiced in Nigeria. Movements of cattle across the study area, as well as the extensive nature of studied pastoralist communities, may have contributed to current observed sero-prevalence, since the state serves as transit route for Fulani nomadic pastoralists on seasonal migrations between the northern parts of the country (that bordered Niger republic, Chad and Cameroon) and southern parts. Movement through bushy vegetation and water bodies that may provide breeding and refuge for mosquitoes as well as contacts with wildlife may have contributed to high RVF antibody detection in the herds. These findings are partly consistent with reports of recent reported outbreaks of RVF in ruminants in the Niger Republic with the origin likely ascribed to animal movements within and from neighbouring countries and likely contacts with wild ruminants during transhumance [64].

There are no previous reports of RVF in the study area, and to our knowledge the disease was never diagnosed clinically in the area. Since no virus isolation was performed in the current study in the current study, it remains to be elucidated whether the cycling virus is a less virulent RVFV strain such as the apathogenic ‘‘clone 13” from the Central African Republic [65,66], or whether acute cases of RVF have been overlooked in the past. Therefore, molecular epidemiological investigation to identify and characterize RVFV in North-central Nigeria is advocated.

Seasonal bias in the present Participatory Epidemiology survey was reduced by conducting exercises across a whole year. Geographical bias was reduced by making specific efforts to cover distant and hard-to-reach pastoralist communities. Subject bias was minimized by giving no special attention to Rift Valley fever even in the introduction of objectives. ‘Dominant-speaker’ among the participants bias was reduced by allowing as many participants as possible to give their views on a certain issue, by also prompting rather silent participants during SSI. Professional team bias was decreased through proper training of the appraisal team. The relatively small sample size of the Key informants in this study was a limitation, but was addressed by allowing as many pastoralists as possible that participated to give their views on all issues. Further, we were limited by lack of full adjustments for the Agro-geographical zones clustering in the designed systematic random sampling of animals used for sero-prevalence as validation. However, the used of central tendency measures would be valuable enough to tolerate the likely imperfections in the confidence intervals.

## Conclusion

The study has shown that Fulani nomadic pastoralists possessed significant existing veterinary knowledge and traditional oral history about RVF and have perceived it to be one of the most important cattle diseases in pastoral communities of Nigeria. Its high impact could be due to absence of surveillance as well as prevention and control strategies in the marginalized rural areas. Our findings in this study have contributed to the understanding of RVF through perceived dynamics and drivers of its occurrence in remote pastoral communities of Nigeria. The perceived relative burden and seasonality of RVF in rural communities, where resources are limited, call for urgent interventions because of its socio-economic and zoonotic importance. The relatively high RVFV IgG sero-positivity without previous reports of RVF and identified risk factors that could predispose to RVF occurrence should serve as early warnings for emergency preparedness for this neglected disease. RVF surveillance system and control programmes that take the identified factors and pathways into consideration will be beneficial to the to the livestock industry in Nigeria, and indeed Africa. The combine use of PE techniques and conventional veterinary methods is essential for ultimate disease surveillance, reporting and control strategies of livestock diseases in Nigeria and therefore recommended. Furthermore, a ‘One Health’ approach is needed to improve efficiency of RVF research, surveillance, prevention and control systems, so as to assure food security and public health in developing countries.

## Supporting information

S1 Fig Proportional piling exercise on diseases at bobi grazing reserve. (TIF)

S2 Fig Proportional piling exercise on risk factors at borgu. (TIF)

S3 Fig Matrix scoring exercise on clinical manifestations at wushishi. (TIF)

S4 Fig Matrix scoring exercise on risk pathways at bosso. (TIF)

S5 Fig Matrix scoring exercise on seasonal calendar at eyagi. (TIF)

S1 Strobe Statement Checklist of items for the participatory epidemiology survey. (DOCX)

S1 Checklist Checklist for identifying and prioritizing Rift Valley fever among other cattle diseases/conditions in nomadic pastoral communities of Niger State. (DOCX)
