# Supplementary figures and images for: Participatory survey of Rift Valley fever in nomadic pastoral communities of North-central Nigeria: The associated risk pathways and factors
Source: PLoS Negl Trop Dis. 2018 Oct 30;12(10):e0006858. doi: 10.1371/journal.pntd.0006858 (PMC6207297; doi:10.1371/journal.pntd.0006858)

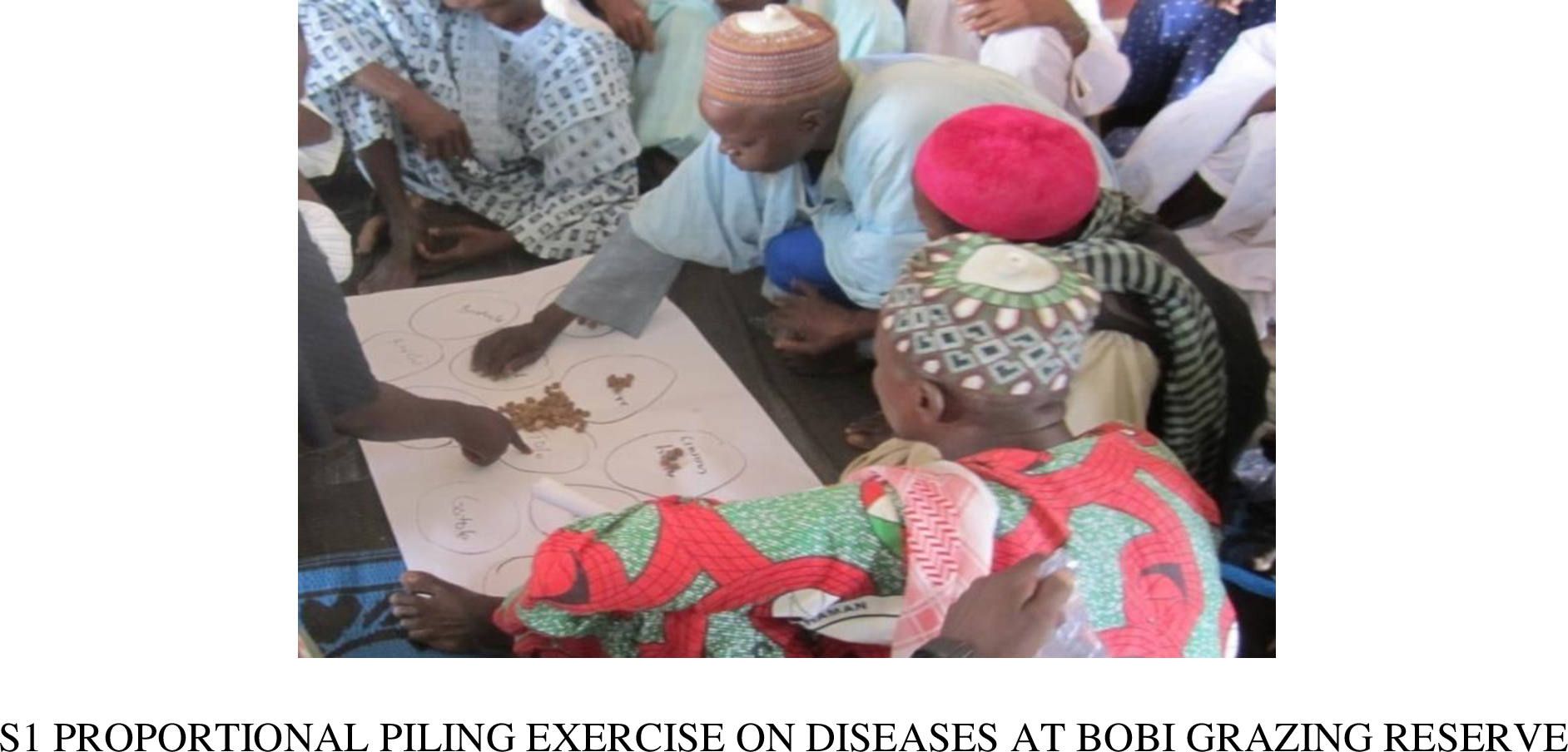

Supplement: S1 Fig — (TIF) [file pntd.0006858.s001.tif]

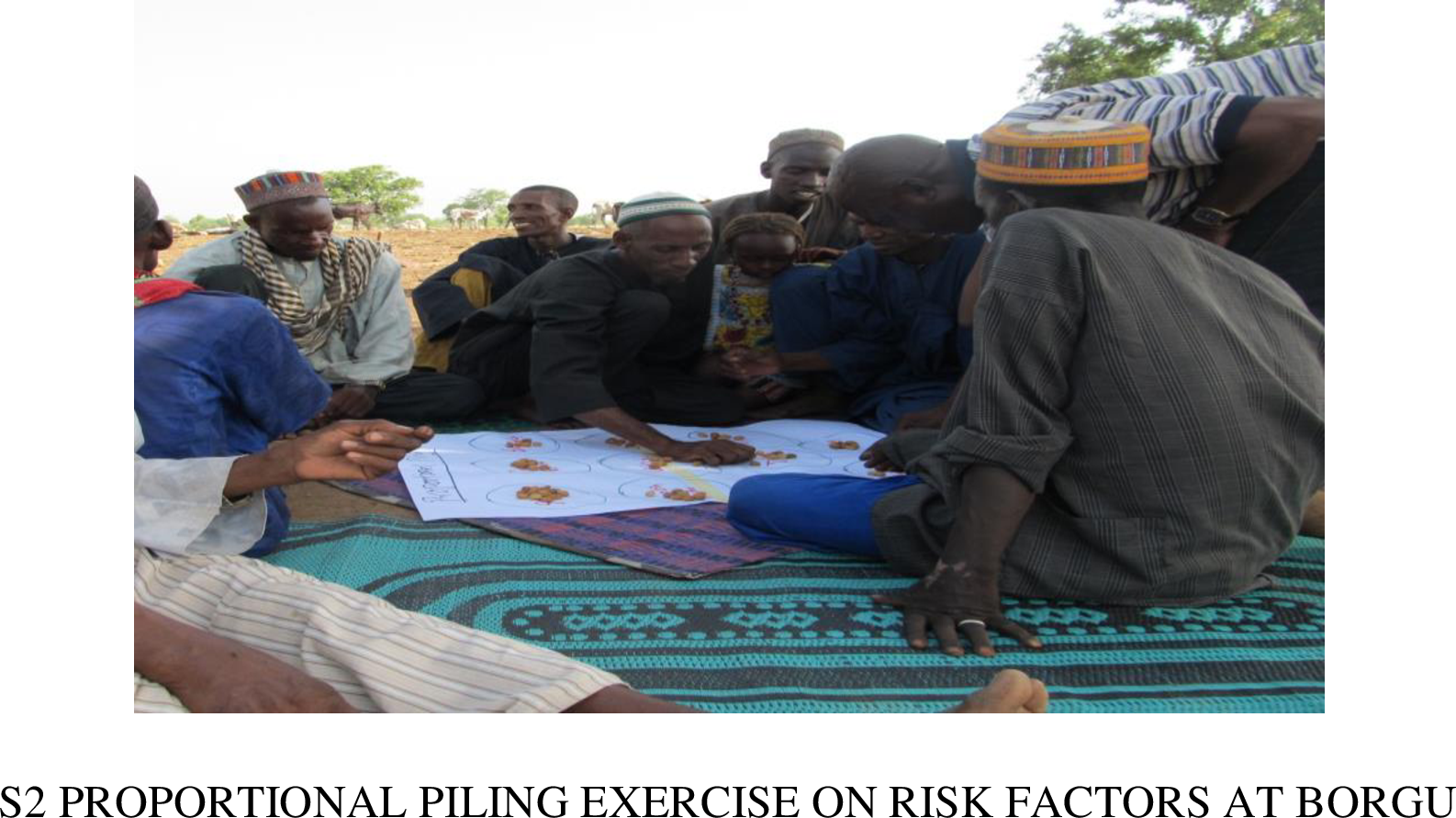

Supplement: S2 Fig — (TIF) [file pntd.0006858.s002.tif]

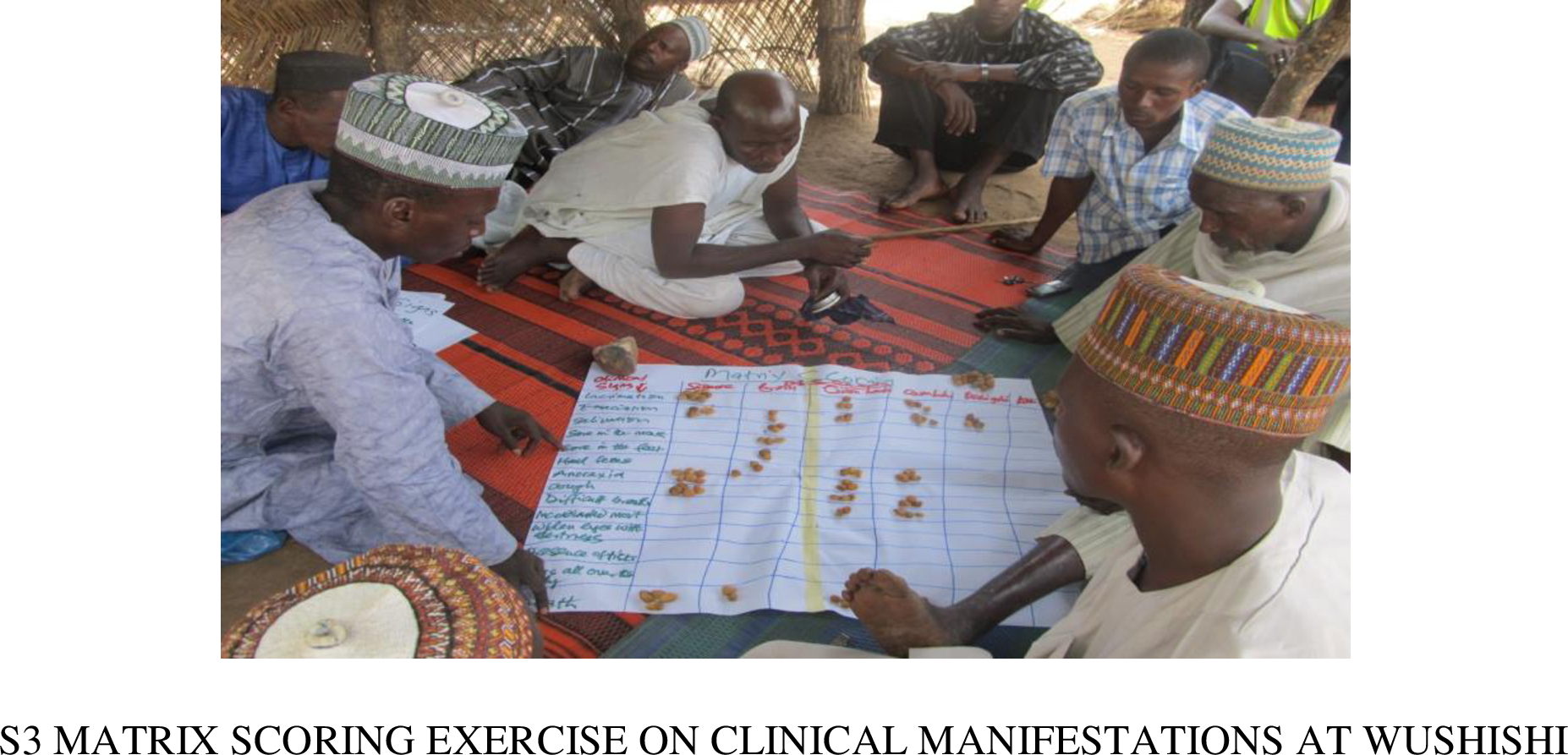

Supplement: S3 Fig — (TIF) [file pntd.0006858.s003.tif]

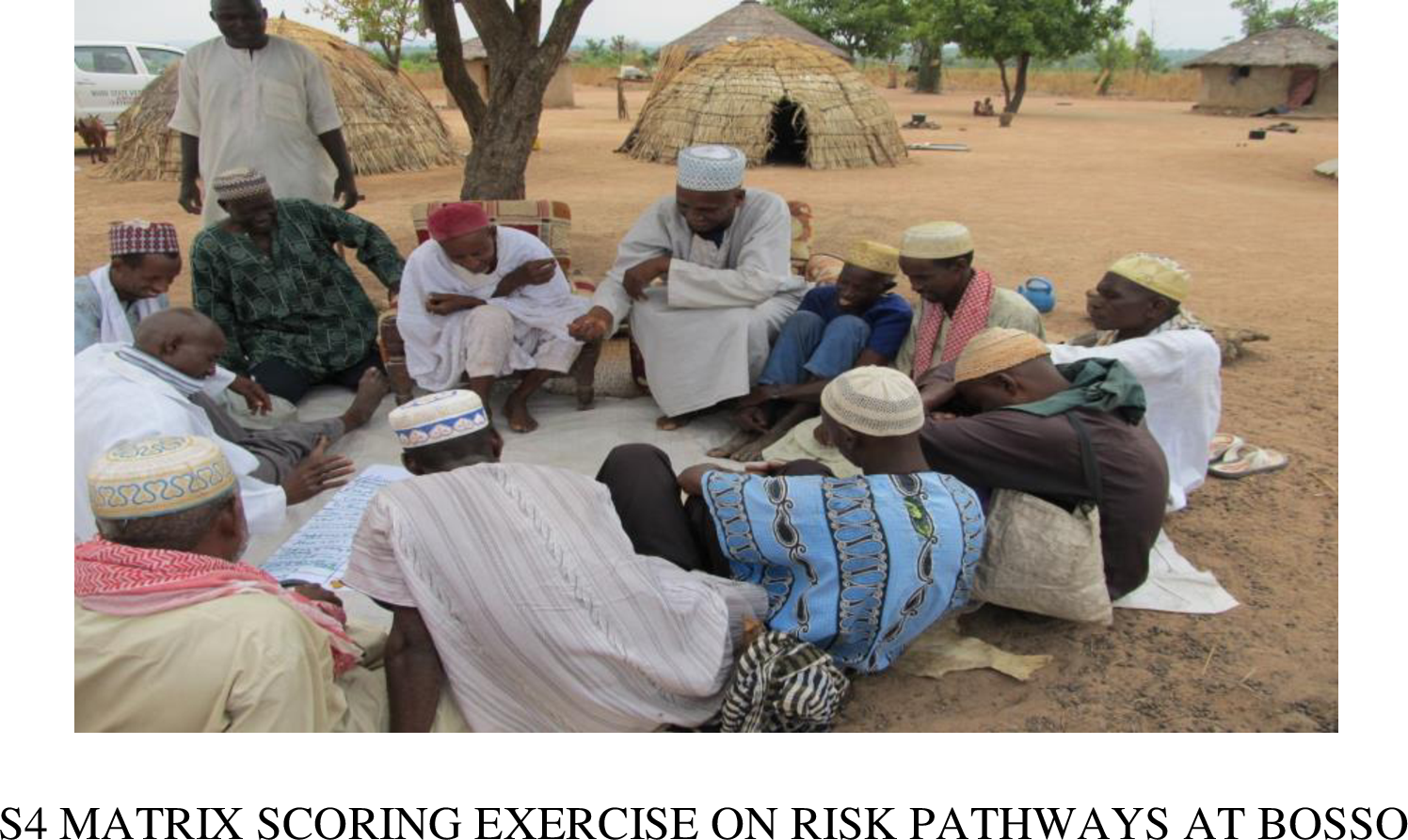

Supplement: S4 Fig — (TIF) [file pntd.0006858.s004.tif]

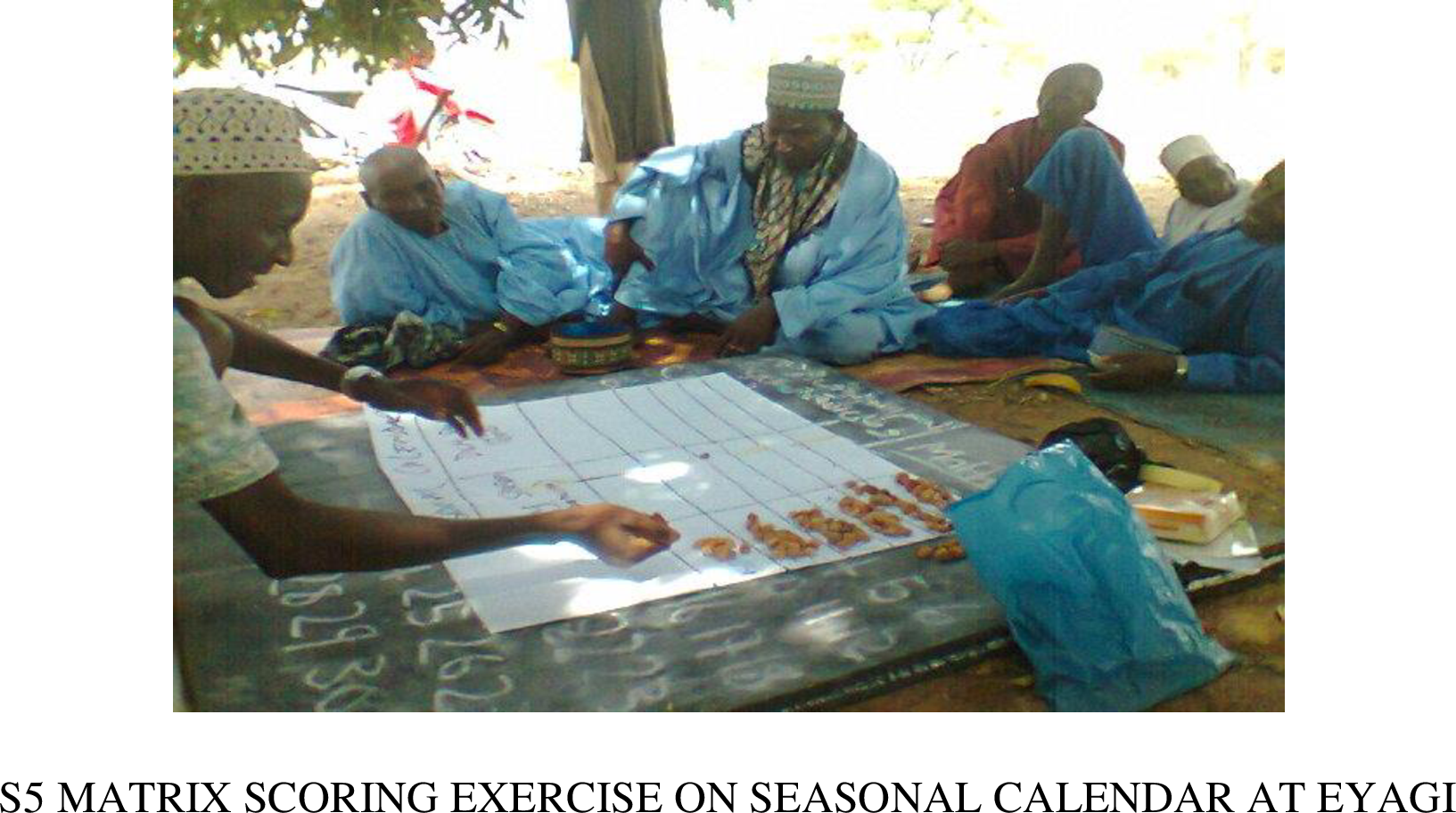

Supplement: S5 Fig — (TIF) [file pntd.0006858.s005.tif]
