# Supplementary material for: Participatory survey of Rift Valley fever in nomadic pastoral communities of North-central Nigeria: The associated risk pathways and factors
Source: PLoS Negl Trop Dis. 2018 Oct 30;12(10):e0006858. doi: 10.1371/journal.pntd.0006858 (PMC6207297; doi:10.1371/journal.pntd.0006858)
Supplement: S1 Strobe Statement — (DOCX) [file pntd.0006858.s006.docx]

**STROBE Statement—Checklist of items for the Participatory Epidemiology survey**

|  | Item No | Recommendation |
| --- | --- | --- |
| **Title and abstract** | 1 | (*a*) Participatory Epidemiology (PE) survey |
|  |  | (*b*) Participatory Epidemiology survey was conducted in Fulani nomadic pastoral communities to assess perceived relative burden and seasonality of RVF in nomadic cattle herds and validate the burden with sero-prevalence; and assess perceived risk factors associated with the disease as well as risk pathways for RVF virus in nomadic pastoral herds of North-central Nigeria using pastoralists’ existing knowledge. Participatory Rural Appraisal tools were used to assess pastoralists’ knowledge about RVF, while systematic randomly sampled cattle had their sera analyzed using c-ELISA. Kendall’s Coefficient of Concordance W statistics and OpenEpi 2.3.1 were used for statistical analyses. The study found that nomadic pastoralists possessed significant existing veterinary knowledge about RVF. There was perceived relative burden and seasonality of RVF in communities. This study finds RVF virus IgG sero-positivity in the study area, and identifies several factors that could predispose to RVF occurrence. |
| Introduction | | |
| Background/rationale | 2 | Rift Valley fever (RVF) is an emerging vector-borne viral zoonotic disease of domestic animals and humans, caused by the Rift Valley fever virus (RVFV). RVFV is transmitted among animals by bites of several species of mosquitoes and by direct contact with body fluids of viremic animals. RVF epidemiology in nomadic pastoral herds is poorly understood in Nigeria, due to paucity of research and surveillance information on the disease. No clinical case of RVF has been formally reported in livestock in Nigeria. Nomadic pastoral communities live in some of the most underdeveloped environments in the world with poor conventional veterinary services and limited basic information on the epidemiology of important livestock diseases. Epidemiological research and disease surveillance in such pastoral areas are difficult because human populations are relatively small and highly mobile with their livestock across large areas. In such situations, conventional approaches to veterinary research and disease surveillance require considerable flexibility and commitment. Given the resource and logistical constraints in such pastoral areas, pastoralists themselves are a valuable source of disease information. |
| Objectives | 3 | The objectives were: to assess perceived relative burden and seasonality of RVF in nomadic cattle herds and validate the burden with sero-prevalence impact; and assess perceived risk factors for the disease and risk pathways for RVFV in nomadic pastoral cattle herds of North-central Nigeria.  Null hypothesized was that nomadic pastoralists do not possessed significant existing veterinary knowledge and traditional oral history about RVF and other cattle diseases and, therefore, they cannot be used for epidemiological investigation of the disease for surveillance and research. |
| Methods | | |
| Study design | 4 | Participatory Epidemiology (PE) survey was conducted using participatory approaches and methods in nomadic pastoral communities domiciled in the state. Also, cross-sectional study was used for sero-positivity validation. |
| Setting | 5 | The study was conducted in Niger State, located at the Southern Guinea savannah in the North-Central geopolitical zone of Nigeria, between latitude 8° 20′ N and 11° 30′ N, and longitude 3° 30′ E and 7° 20′ E, between January and December 2015. PE data were collected using participatory rural appraisal tools of semi-structured interview, checklist, key informants, proportional piling, matrix scoring, seasonal calendar and triangulation. |
| Participants | 6 | (*a*) The target populations were Fulani nomadic pastoralists, who are seasonally mobile, with scattered herds of local breeds of cattle (Bunaji, Rahaji and Bokoloji), domiciled in remote areas of the state during the study period. Fulani nomadic pastoralist community leaders were purposively selected because they were considered to be more knowledgeable than others on animal health and production management. Systematic random sampling method was used to select animals used for sero-validation. |
| Variables | 7 | In the present study, predictors were the pastoralists (judges), outcomes were existing knowledge and traditional oral history about relative burden and seasonality of RVF and other important cattle diseases, exposures were the risk factors that predisposed to RVF occurrence in herds e.g. mosquito bites, infected aborted foetuses etc. |
| Data sources/ measurement | 8* | For relative burden and perceived risk factors of the disease, data were obtained from proportional piling exercises. For clinical manifestations, risk pathways and seasons of RVF occurrence, data were from matrix scoring exercises. Methods of assessments (measurement) were by the semi-quantitative piles and scores. Triangulation was used to compare the semi-quantitative piles and scores and also between the semi-quantitative and quantitative piles and scores from the communities. |
| Bias | 9 | Seasonal bias was reduced by conducting exercises across a whole year. Geographical bias was reduced by making specific efforts to cover distant and hard-to-reach pastoral communities. Subject bias was minimized by giving no special attention to Rift Valley fever even in the introduction of objectives. ‘Dominant-speaker’ among the participants bias was reduced by allowing as many participants as possible to give their views on a certain issue, by also prompting rather silent participants during SSI. |
| Study size | 10 | For the PE, study size was conveniently determined. For the sero-prevalence study, sample size was computed for finite population correction factor N using the **Open Source Epidemiologic Statistics for Public Health (**OpenEpi) 2.3.1 software, with power set at 50%, and 10% margin of error at 95% confidence level. A sample size of 97 nomadic cattle was computed. |
| Quantitative variables | 11 | A competitive Enzyme Linked Immunosorbent Assay (c-ELISA) was used for detecting RVFV-specific antibodies according to the manufacturer’s instructions. |
| Statistical methods | 12 | (*a*) The Kendall’s Coefficient of Concordance W statistic, a non-parametric statistics was used for ranking and scoring. Descriptive statistics of frequency and proportion were used to describe obtained serological data and analyzed using the Open Source Epidemiologic Statistics for Public Health (OpenEpi) version 2.3.1. All analyses were performed at 95% confidence level. |
|  |  | (*b*) None |
|  |  | (*c*) The exercises were participatory oriented, and therefore there was no issue of missing data to address. |
|  |  | (*d*) The analytical method used was the Kendall’s Coefficient of Concordance W statistic and used applied ranking to assessed the scores. |
|  |  | (*e*) None. |
| Results | | |
| Participants | 13* | (a) 27 Key informants were potentially eligible, examined for eligibility, confirmed to be eligible, and were included in the study. |
|  |  | (b) All participated. |
|  |  | (c) None |
| Descriptive data | 14* | (a) All were Fulani nomadic pastoralists, males and females, leaders of their respective pastoralist communities, and were considered to more knowledgeable about livestock health and management. |
|  |  | (b) None |
| Outcome data | 15* | Five i.e. relative burden, clinical manifestations, risk pathways, seasonal occurrence, and sero-positivity. |
| Main results | 16 | (*a*) All estimates were unadjusted at precision of 95% confidence interval. |
|  |  | (*b*)None |
|  |  | (*c*) None |
| Other analyses | 17 | None |
| Discussion | | |
| Key results | 18 | The relative burden of RVF among other cattle diseases was 8.3%. RVF occurs more in late rainy season (5.3). Entry pathway of RVF agent into nomadic pastoral herds were: presence of RVFV infected mosquitoes (tiny biting flies) (7.9), presence of infected cattle in herds (8.4), and contacts of herd with infected wild animals at grazing (10.1). Spread pathway of RVFV in the herds were bites of infected mosquitoes at the grazing and watering points (5.1), contacts with infected aborted foetuses and fluids at the herd settlements and grazing areas (7.8), and contaminated pasture with aborted foetuses and fluids (9.7). Mosquitoes’ bites had high risk consequence of 9.9, while contaminated pasture with infected aborted foetuses and fluids had low risks with 2.6 scores. Obtained 11.3% (11/97; 95% CI: 6.1, 18.9) sero-positivity was used for validation. |
| Limitations | 19 | The relatively small sample size of the Key informants in this study was a limitation, but was addressed by allowing as many pastoralist participants as possible to give their views on all issues. We were also limited by lack of full adjustments for the Agro-geographical zones clustering in the designed systematic random sampling of animals used for sero-prevalence as validation. However, the used of central tendency measures would be valuable enough to tolerate the likely imperfections in the confidence intervals. |
| Interpretation | 20 | The study has shown that Fulani nomadic pastoralists possessed significant existing veterinary knowledge about RVF and have perceived it to be one of the most important cattle diseases. Its high impact could be due to absence of effective approach to surveillance as well as prevention and control strategies in the marginalized rural areas. The study has contributed to the understanding of relationship between RVF dynamics and drivers in remote pastoral communities of Nigeria. There was a relatively high RVF virus IgG sero-positivity without previous reports of RVF , and several factors that could predispose to its occurrence were identified. These can serve as early warnings for emergency preparedness for this neglected disease. The use of Participatory Epidemiology approach is needful in active surveillance of livestock diseases in nomadic herds domiciled in highly remote areas as previously reported from similar studies. |
| Generalisability | 21 | The Fulani nomadic pastoralists provided more detailed and accurate clinical descriptions of important diseases affecting their cattle, including RVF. Pastoralists called Rift Valley fever *Gabi-gabi,* denoting disease associated with high fever, diarrhoea, lacrimation, nasal discharge, neurological disorder, abortions in pregnant cows sand sudden death in newborns (calves). This is the general clinical description of the disease and suggests that the PE approaches can detect RVF occurrences earlier by taking advantage of pastoralists’ observations through integration of active syndromic surveillance, such as participatory disease surveillance (PDS) geared to the level of outbreak probability as reported by conventional studies. |
| Other information | | |
| Funding | 22 | The present study was funded mainly by the corresponding author as part of his PhD works, from 30% monthly contributions of his salary for over a period of three years. |

*Give information separately for exposed and unexposed groups.

**Note:** An Explanation and Elaboration article discusses each checklist item and gives methodological background and published examples of transparent reporting. The STROBE checklist is best used in conjunction with this article (freely available on the Web sites of PLoS Medicine at http://www.plosmedicine.org/, Annals of Internal Medicine at http://www.annals.org/, and Epidemiology at http://www.epidem.com/). Information on the STROBE Initiative is available at www.strobe-statement.org.
