# Supplementary material for: Participatory survey of Rift Valley fever in nomadic pastoral communities of North-central Nigeria: The associated risk pathways and factors
Source: PLoS Negl Trop Dis. 2018 Oct 30;12(10):e0006858. doi: 10.1371/journal.pntd.0006858 (PMC6207297; doi:10.1371/journal.pntd.0006858)
Supplement: S1 Checklist — (DOCX) [file pntd.0006858.s007.docx]

**Checklist for identifying and prioritizing Rift Valley fever among other cattle diseases/conditions in nomadic pastoral communities of Niger State**

1. Introduction of Appraisal team as animal health workers conducting a research
2. Identification of the participants led by key informants
3. Establish the breeds of cattle kept by the pastoralists
4. Establish the husbandry management system practiced by the pastoralists
5. Identify and describe the most important cattle disease problems that have affected herds in the last ten (10) years
6. Description of clinical manifestations of disease problems mentioned in 5
7. If RVF is mentioned, probing to get more information about it
8. Identify and describe risk factors that can predispose to RVF occurrence in animals
9. Identify and describe risk pathways for entry of RVF into herds and consequences (effects) on animals when established
10. Establish the influence of season (if any) on RVF occurrence in herds
11. Proportional piling exercises on identified important cattle diseases/conditions
12. Matrix scoring exercises on identified clinical manifestations of identified diseases/conditions
13. Proportional piling exercises on RVF risk factors
14. Proportional piling exercises on RVF risk pathways
15. Matrix scoring exercises on seasonal calendar of RVF occurrence
16. Triangulation (validation) of exercises results
